# Continuous intraoperative neuromonitoring of the recurrent laryngeal nerve by eliciting the laryngeal adductor reflex (LAR-CIONM)

**DOI:** 10.1515/iss-2021-0008

**Published:** 2022-07-04

**Authors:** Catherine F. Sinclair, Maria J. Tellez

**Affiliations:** Department of Otolaryngology Head and Neck Surgery, Mount Sinai West Hospital, New York, NY, USA; Department of Neurosurgery, Mount Sinai West Hospital, New York, NY, USA

**Keywords:** continuous, larynx, neurolaryngology, neuromonitoring, recurrent laryngeal nerve, reflex, thyroid, vocal cord palsy

## Abstract

The laryngeal adductor reflex (LAR) is a life-sustaining airway protective mechanism that serves to shield the lower airways from inhaled foreign bodies. Over the past half century, the LAR has been extensively investigated and its dysfunction has been linked to far-ranging pathologies, from dysphagia to sudden infant death syndrome. Over the past 6 years, specific electromyographic waves in the LAR response have been used to devise a methodology for monitoring the vagus and recurrently laryngeal nerves during surgical procedures. This methodology involves continuous intraoperative neuromonitoring of the laryngeal adductor reflex and isthus termed ‘LAR-CIONM’. In this review paper, the physiology of the LAR will be summarized as it relates to LAR-CIONM and the technique of LAR-CIONM will be described. Applications of this technique and published outcomes of LAR-CIONM will be highlighted.

## Introduction

The laryngeal adductor reflex (LAR) is a life-sustaining airway protective mechanism that serves to shield the lower airways from inhaled foreign bodies [1]. It is particularly essential in humans due to the relatively inferior position of the larynx within the neck and thus the greater common shared space between respiratory and deglutition tracts [2]. The LAR is a bilateral reflex and has afferent, central and efferent components. It is initiated by supraglottic mucosal receptor stimulation [3]. Afferent information is carried via the ipsilateral internal branch of the superior laryngeal (iSLN) and vagus nerves to the brainstem. After synapsing in the brainstem, efferent information is carried by the vagus and recurrent laryngeal (RLN) nerves to the laryngeal adductor muscles–the lateral cricoarytenoid and thyroarytenoid muscles [1, 4]. Contraction of these muscles results in bilateral vocal fold adduction, effectively shutting the valve between the upper and lower airways.

The reflex consists of two main electromyographic (EMG) waves as recorded from laryngeal adductor muscles–an early R1 component with an approximate latency of 20 ms (ms) and a later R2 component of approximate latency 50–60 ms [5], [6], [7], [8], [9], [10]. These R1 and R2 components are distinct from the R1/R2 responses described by The International Neural Monitoring Study Group [4] which refer to the laryngeal compound muscle action potentials (CMAP) evoked upon direct stimulation of the RLN at the start (R1) and end (R2) of a neck endocrine surgery. CMAP responses differ physiologically from LAR responses and are not directly comparable in terms of characteristics such as latency and amplitude.

Over the past half century, the LAR has been extensively investigated and its dysfunction has been linked to far-ranging pathologies, from dysphagia to sudden infant death syndrome [11, 12]. Until recently, it was thought that the R2 component was the predominant contractile wave of the reflex and that this was abolished in humans under altered states of consciousness, including general anesthesia. However, over the past 6 years, a series of human studies has re-evaluated the intrinsic properties of the LAR and contradicted many prior beliefs regarding this life-sustaining reflex. These investigations have enabled the LAR to be used as a s*continuous* intraoperative neuromonitoring technique (CIONM) for the RLN and vagus nerves, thus this methodology is termed ‘LAR-CIONM’ [6, 9, 10]. This paper will begin by briefly summarizing some of these novel physiologic findings as they relate to LAR-CIONM and subsequently describe the technique, uses and outcomes of LAR-CIONM.

### LAR physiology

Early studies on the LAR suggested that it was absent under general anesthesia [1, 13]. These studies were done on animals and humans using inhalational anesthetic agents with the findings that the R2 response was completely abolished and the R1 response lost its bilateral form, becoming ipsilateral. In *awake* humans, early studies suggested that the R1 component was ipsilateral whereas the R2 component was bilateral and strong. The summation of these findings led to the suggestion that the R2 component was the predominant electrical event causing reflex vocal fold closure in response to supraglottic irritation.

Inhalational agents suppress reflex responses and these effects can be eliminated by the use of total intravenous anesthesia (TIVA). As such, when the LAR was initiated by electrical supraglottic mucosal stimulation under TIVA, the R1 response was robustly bilateral and caused vocal fold contraction even in the absence of a visible R2 wave, suggesting that the R1 response plays an active role in initiation of vocal fold contraction [5], [6], [7], [8]. Similarly, when the reflex was elicited by transcutaneous iSLN stimulation in *awake* humans, a robust bilateral R1 response was again obtained, validating the findings under TIVA regarding the role of the R1 response in LAR initiation.

Mucosal receptors activating the LAR response can be stimulated by many methods, including touch, chemicals and electricity. The greatest density of LAR-activating receptors is located posteriorly in the interarytenoid area and over the medial surface of the arytenoid cartilages. Stimulation of this area elicits the most robust reflex response [7]. The EMG endotracheal tubes used intraoperatively by most neck endocrine surgeons contain surface electrodes designed to *record* EMG activity resulting from vocal fold adduction. However, when positioned correctly, these electrodes can also be used to electrically stimulate the supraglottic mucosa and activate the LAR. This forms the basis for LAR-CIONM as discussed subsequently.

### The evolution and technique of LAR-CIONM

In 2015, it was determined that a robust bilateral R1 LAR response could be obtained under total intravenous anesthesia [5, 6]. This R1 response persisted for the duration of a surgical procedure without fatiguing and decrements in the amplitude of the response mirrored decrements in the CMAP response which occurred secondary to nerve irritation/injury. Unlike prior methods of continuous vagal IONM which recorded compound muscle action potentials (CMAP) from the laryngeal adductor musculature using a circumferential vagus nerve electrode, LAR-CIONM required only an EMG endotracheal tube to provide continuous data on nerve integrity. The response could be elicited prior to skin incision thereby giving a true baseline value before any dissection around the nerves was initiated. The bilateral nature of the response allowed the LAR waveforms from the RLN not-at-risk to serve as a control against endotracheal tube motion or rotation. All of these factors made LAR-CIONM a very appealing methodology to pursue as an alternative form of CIONM for the vagus and recurrent laryngeal nerves.

### Technique

**Figure 1:**
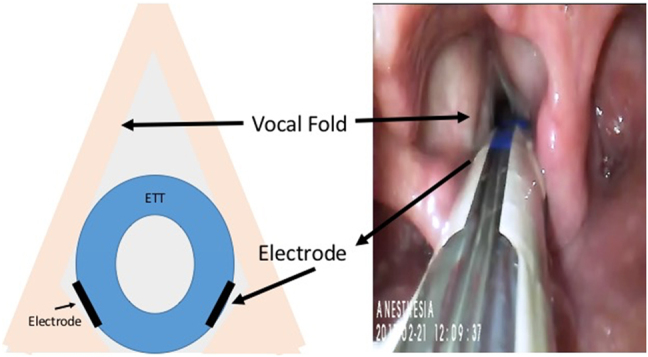
Endotracheal tube positioning for LAR-CIONM with electrodes touching the medial arytenoid mucosa.

After induction of general anesthesia, the patient is intubated with a EMG endotracheal tube that has electrodes positioned such that they come into direct contact with the posterior aspects of the right and left vocal folds (Figure 1). Following initial intubation, tube position is rechecked after the patient is properly positioned for the neck surgery, and then secured to the mouth using an oral endotracheal tube (ETT) fastener (Anchor-FastTM, Libertyville, IL, USA) and tape. Anesthesia is maintained with propofol (100–150 µg/kg/min) and remifentanil or sufentanil (1 µg/kg/min). No inhalational agents are used. Short-acting muscle relaxants are used only for tracheal intubation and avoided thereafter.

The LAR is elicited by electrical stimulation of laryngeal mucosa using tube electrodes on the side contralateral to the surgical field. A single-stimulus (0.1–1 ms duration) or short train of 2–3 pulses (1–2 ms interinterval stimulus) at intensity ≤10 mA are applied at 0.7–1.0 Hz repetition rate throughout the surgery. LAR responses are recorded on tube electrodes located ipsilateral to the surgical field for monitoring the RLN at risk.

### Choice of side for stimulation vs. recording during LAR-CIONM

**Figure 2:**
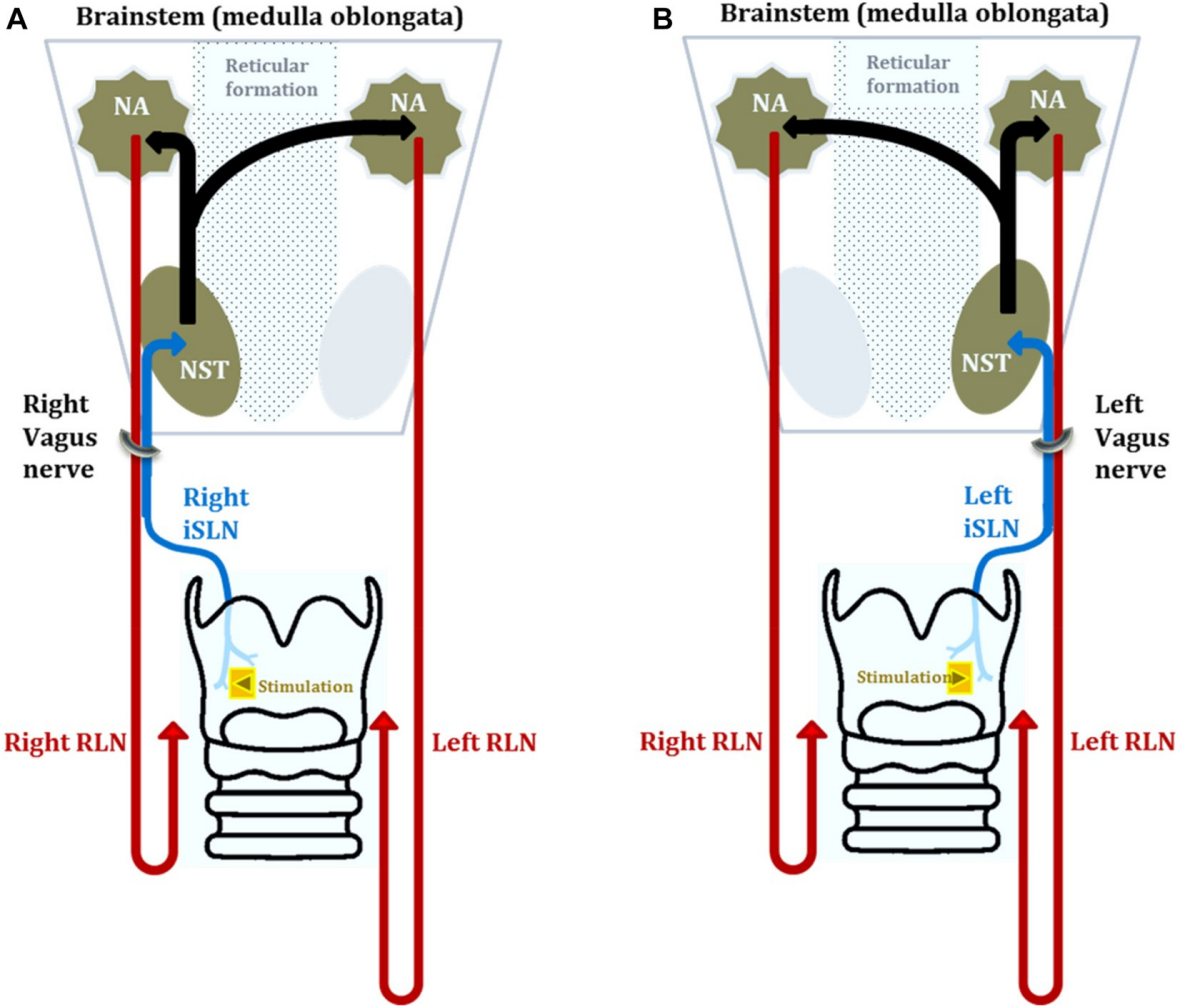
(A) Stimulation of the right iSLN during a surgery on the left neck and recording cR1 potentials from the left vocal fold gives a measure of the motor integrity of the left vagus/RLN pathway. (B) Stimulation of the left iSLN during a surgery on the left neck and recording cR1 potentials from the right vocal fold gives a measure of the sensory integrity of the left vagus/RLN pathway.

Depending on whether supraglottic mucosa on the ipsilateral or contralateral side (relative to the operative field) is stimulated, sensory (iSLN) or motor (RLN) functions of the vagus nerve can be monitored. For example, in the case of a left sided neck lesion, electrical stimulation of the laryngeal mucosa on the side contralateral to the tumor (i.e., the right supraglottic mucosa) carries sensory information to the brainstem via the right iSLN, and activation of motor neurons located in bilateral nucleus ambiguui will follow. By recording the contraction of vocal muscles supplied by the left RLN in the right larynx (ipsilateral to the tumor), the LAR provides data on the motor integrity of the vagus nerve at risk (termed ‘motor-LAR’) (Figure 2A). Conversely, stimulation of supraglottic laryngeal mucosa ipsilateral to the operative field with recording of right sided RLN-induced vocal fold contraction provides sensory information of the left vagus nerve at risk (termed ‘sensory-LAR’) (Figures 2B).

### LAR-CIONM in neck endocrine surgery

The proximity of the laryngeal nerves to the thyroid gland places them at risk of injury in all neck endocrine procedures. The ability to monitor their function continuously allows the surgeon to recognize and prevent slowly evolving nerve injuries (such as those due to traction or compression) by technique modification according to IONM feedback obtained.

**Figure 3:**
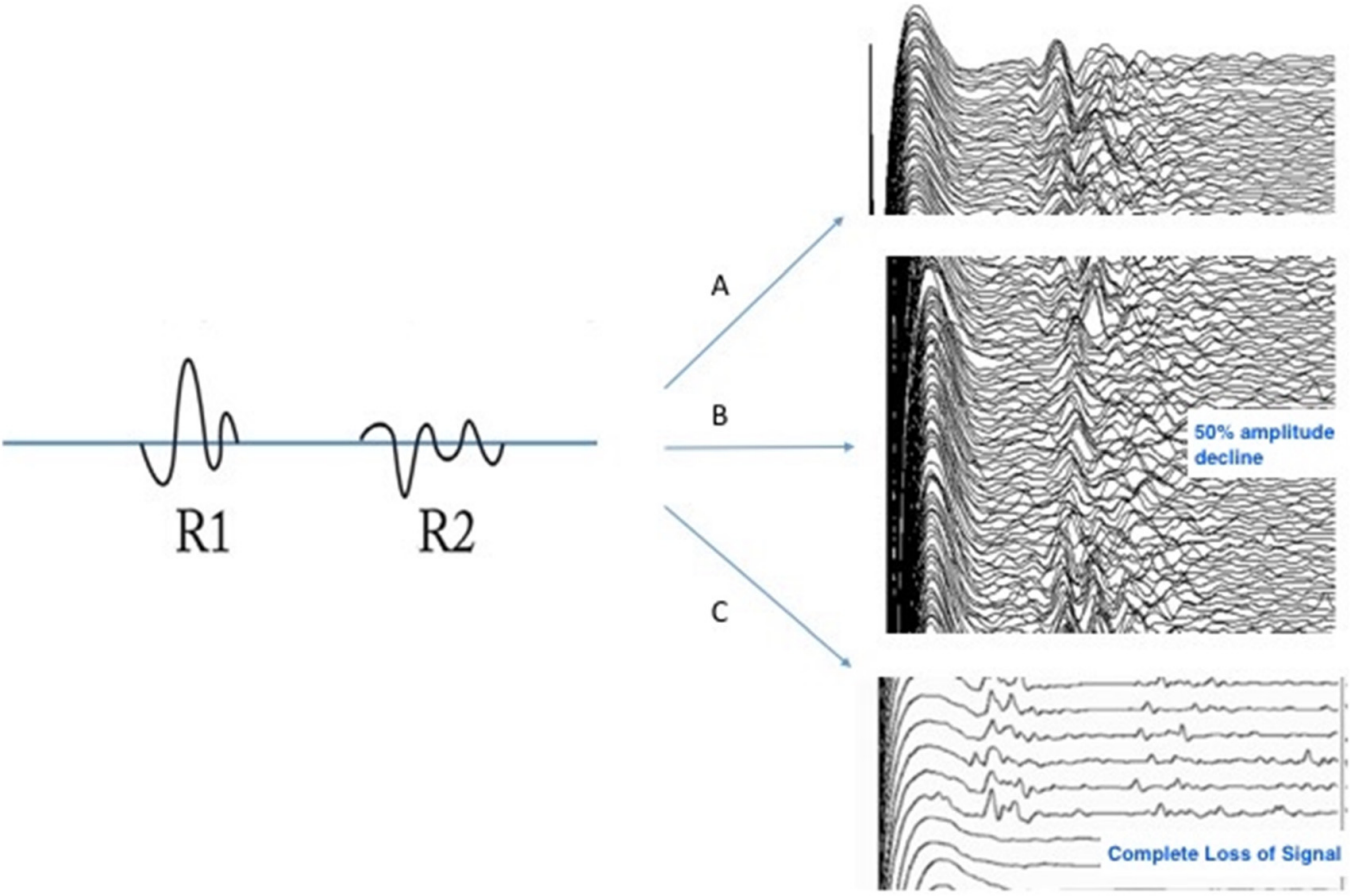
Three different outcomes for LAR-CIONM. A. Normal baseline traces showing no significant amplitude declines in the R1 component of the LAR. B. Electromyographic traces showing a 50% amplitude decline in the R1 component of the LAR which recovered to baseline upon release of tissue traction. C. Sudden loss of signal in the LAR response due to proximate cautery.

Until recently, the only available CIONM technique involved the placement of a ring electrode around the vagus nerve to stimulate vagal motor fibers continuously (Table 1) [14], [15], [16], [17], [18], [19], [20]. This type of direct nerve stimulation elicits compound muscle action potentials (CMAP) in laryngeal muscles which are recorded on endotracheal tube (ETT) surface electrodes. The position of vagus nerve is variable, most commonly located posterior and lateral to the common carotid artery but sometimes located between the carotid and jugular or anteriorly [21]. Such deep positioning can make dissection of the nerve difficult and has been reported to occasionally lead to vagus nerve injury. LAR-CIONM does not require a vagal electrode and is preferable when positioning of this electrode is difficult due to factors such as small incision size, deep neck anatomy, and remote access approaches to the thyroid.

**Table 1:** A summary of vagal CIONM methodologies to date.

31. Author	Targeted structure	CIONM methods	Invasive	Motor monitoring	Sensory monitoring	Brainstem (medulla) monitoring	Setting (number of nerves)
Lamade W, 1997	RLN	Trans-tracheal by endotracheal tube	No	Yes	No	No	Experimental
Smith DB, 1989	RLN	Double-ballooned endotracheal tube and pressure transducer system	No	Yes	No	No	Experimental
Lamade W, 2007	VN	Vagal nerve cuff electrode	Yes	Yes	No	No	Experimental
Ulmer C, 2008							*In-vivo* (n=19)
Schneider R, 2009							*In-vivo* (n=78)
Chiang FY, 2015	RLN	Stimulating dissecting instruments	No	Yes	No	No	*In-vivo* (n=168)
Zhang D, 2019							Experimental
Sinclair CF, 2020	VN and medulla	Endotracheal tube electrodes	No	Yes	Yes	Yes	*In-vivo* (n=205)
Tellez MJ, 2020							*In-vivo* (n=53)
Wu CW, 2013	VN	Hand probe continuous stimulation on the carotid sheath	No	Yes	No	No	*In-vivo* (n=376)
Zhang D, 2018	VN	Percutaneous stimulation	Yes	Yes	No	No	*In-vivo* (n=277)
Suh I, 2016	VN	Transcutaneous stimulation	Yes	Yes	No	No	*In-vivo*(n=4)

In 2016, the contralateral R1 component of the LAR was discovered and developed as a novel system of CIONM. An initial feasibility study of LAR-CIONM with 134 nerves-at-risk showed that LAR vocal fold waveforms are distinct from CMAP waveforms, demonstrating that LAR-CIONM is highly sensitive to nerve traction or compression [9]. Intraoperative decreases in LAR response amplitude during periods of nerve stress mirrored decreases in CMAP responses. Irreversible LAR amplitude loss of signal predicted postoperative vocal fold paralysis (Figure 3). Data from this study were used to create unique LAR-CIONM warning criteria for nerve injury. A recent prospective case-control study of 335 nerves at risk used historical controls and showed a reduction in temporary vocal fold palsy when LAR-CIONM was used compared with intermittent IONM alone. Permanent nerve palsy rates were constant [10]. Future multi-center studies are necessary to better define warning criteria for LAR-CIONM loss of signal.

It is important to note that, in all neck endocrine surgeries monitored using LAR-CIONM, intermittent IONM is also utilized via a handheld probe to directly stimulate the RLN and vagus nerves. LAR-CIONM does not assist in nerve localization or mapping. Intermittent IONM does not assist in continuous functional nerve assessment. As such the two techniques are complementary and best results are obtained when both techniques are utilized for each patient.

Use of the LAR in neck endocrine procedures may also allow nuances in laryngeal behavior to be properly diagnosed and managed. For instance, normal postoperative vocal fold motility while the patient says “eee” under laryngoscope examination does not exclude laryngeal hypoesthesia and therefore does not rule out a higher risk of aspiration. A decline in the intraoperative LAR amplitude could help understand why a patient coughs on swallowing liquids, even if gross vocal fold mobility appears normal. As such, LAR-CIONM provides information that cannot be gleaned from simple assessment of voice and speech along using normal vocal fold mobility as a marker for normal function and may enable a more holistic assessment of laryngeal neurophysiology.

### Additional applications of LAR-CIONM

To date, LAR-CIONM has been successfully applied to vagal schwannoma surgeries, posterior fossa surgeries and cervical spine (anterior approach) surgeries. A brief summary of these applications follows:

### Vagal schwannoma excision

Schwannomas are benign tumors arising from neurolemmocytes. They become symptomatic when the tumor grows large enough to disrupt vagal function. Presenting symptoms can include hoarseness, dysphagia, aspiration and a neck mass. Simple excision of symptomatic, enlarging vagal schwannomas results in complete vocal fold palsy due to vagal nerve fiber disruption plus effects on swallowing and laryngopharyngeal sensation. As such, tumor enucleation is now the preserved surgical management option. Enucleation leaves tumor capsule *in situ* but can preserve the vagus nerve fibers running within that capsule. Unfortunately, visual preservation of nerve fibers does not equate to functional preservation [22], [23], [24], [25].

Recently, the LAR was utilized to map nerve fibers–*motor and sensory*–on the surface of high vagal schwannomas [26]. Motor fibers were mapped using a handheld probe applied directly to the nerve fibers–this elicited a CMAP potential in vocal muscles. Sensory fibers from the internal superior laryngeal nerve were also mapped using a handheld probe which triggered a laryngeal adductor response. This was the first time ever the functional activity or peripheral sensory vagal nerve fibers could be actively assessed intra-operatively. Afferent information was carried by these sensory fibers to the brainstem then back down the motor fibers of the vagus nerve, resulting in a bilateral LAR response. Thus, even if the ipsilateral vocal fold was paralyzed preoperatively due to tumor-induced motor fiber disruption, the sensory fibers could still be mapped by measuring the contralateral vocal fold contractile response. In addition to localization and mapping of functional nerve fibers, LAR-CIONM was utilized at all other stages of the dissection to ensure that nerve functional activity was preserved, especially during initial mobilization of the tumor when traction injury can commonly occur.

### Posterior fossa surgery

There have been a series of papers released in the past two years regarding use of LAR-CIONM during posterior fossa surgeries, in both children and adults. In these cases, LAR-CIONM was utilized to monitor for functional changes during brainstem tumor exposure and dissection. Elicitation of the LAR by direct stimulation of brainstem vagal nuclei with resultant vocal fold contraction was used to confirm functional viability of LAR central pathways. Loss of LAR-CIONM signal correlated with postoperative hoarseness and dysphagia. The largest of these series, a multi-institutional study of 53 patients, showed that permanent significant decrement or loss of the LAR R1 response correlated with postoperative laryngeal dysfunction and predicted motor and sensory dysfunction of the vagus nerve and reflexive medullary pathways. By contrast, a significant decrement in LAR R1 amplitude that recovered after a timely surgical adjustment prevented irreversible damage [27].

### Anterior approach cervical spine surgery

During an anterior approach to the cervical spine, different branches of the vagus nerve can be at risk of injury depending on the pathologic cervical level. There are three main periods during a cervical spine surgery when these nerves are at greatest risk: 1. during initial neck dissection to expose the prevertebral plane; 2. during pharyngeal mobilization off the pre-vertebral fascia; and 3. during placement of neck retractors. These retractors must hold the pharynx, larynx and esophagus to one side of midline to allow access to the vertebral bodies. This positioning of neck viscera can cause traction related injury to vagus nerve branches.

The recurrent laryngeal nerve is at greatest risk during low approaches (C5/6 and C6/7) as it runs in the tracheoesophageal groove to the larynx. The purely sensory iSLN is at greatest risk during high approaches (C2/3, C3/4) as it curves anteriorly to enter the larynx via the thyrohyoid membrane at approximately C3/4. LAR-CIONM allows both vagal branches to be monitored depending on the level of approach. For iSLN monitoring, the LAR response is generated by mucosal laryngeal stimulation ipsilateral to the side of surgical approach to ensure that afferent sensory impulses travel up the nerve-at-risk. For RLN monitoring, mucosal stimulation occurs contralateral to the operative field. Any functional disruption of nerve fibers during initial approach can thereby be identified.

### Anesthesetic implications

The LAR is stable and bilateral under total intravenous anesthesia. However, under inhalational halogenated anesthetic agents, the LAR is a weakness. This concept has important implications for managing general anesthesia and preventing anesthesia-related aspiration events. Preservation of the LAR could be desirable for patients at high risk of aspiration, such as following emergency intubation where preoperative fasting was impossible. As such, avoidance of any inhalational anesthetic agents is mandatory. Conversely, patients at high risk of laryngospasm where elimination of the LAR is desirable would benefit from administering inhalational agents immediately before extubation with or without topical anesthesia applied directly to supraglottic mucosa, especially in the interarytenoid region where receptor density is greatest [7].

## Conclusions

LAR-CIONM is a novel methodology to continuously monitor vagus, recurrent laryngeal, and internal superior laryngeal nerve function. It has wide applicability to a variety of surgeries and is starting to be used by surgical groups worldwide. For optimal results, LAR-CIONM should be used in combination with intermittent IONM as LAR-CIONM cannot assist in nerve localization or mapping whereas intermittent IONM can provide no continuous assessment of nerve functional integrity. Intermittent IONM thus has limited ability to prevent nerve injury. High surgeon responsiveness to the feedback obtained from CIONM is essential if this technique is to be used successfully to prevent slowly evolving forms of nerve injury. Future studies in larger populations will help to refine existing warning criteria for LAR-CIONM and clarify its utility in surgeries other than neck endocrine procedures.

## Supplementary Material

Supplementary Material
