# Supplementary material for: Continuous intraoperative neuromonitoring of the recurrent laryngeal nerve by eliciting the laryngeal adductor reflex (LAR-CIONM)
Source: Innov Surg Sci. 2022 Jul 4;7(3-4):79–85. doi: 10.1515/iss-2021-0008 (PMC9742263; doi:10.1515/iss-2021-0008)
Supplement: Supplementary file 1 — Supplementary Material [file j_iss-2021-0008_suppl.pdf]

Reviewer Assessment

C.F. Sinclair & M.J. Tellez: Continuous intraoperative neuromonitoring of the recurrent laryngeal nerve by eliciting the laryngeal adductor reflex (LAR-CIONM)

Reviewers' Comments to Original Submission

Reviewer 1: anonymous

Date received: 09-May-2021
Reviewer recommendation: **Return to author for minor modifications**
Reviewer overall scoring: **Medium**

Assessment Form scores: 5 = High/Yes; 3 = Medium/Adequate; 1 = Low

Is the subject area appropriate for the journal		4			
Does the title clearly reflect the paper's content?		4			
Does the abstract clearly reflect the paper's content		4			
Do the keywords clearly reflect the paper's content?		4			
Does the introduction present the problem clearly?			3		
Are the results/ conclusions justified?			3		
How comprehensive and up-to-date is the subject matter presented?		4			
How adequate is the data presentation?				2	
Are units and terminology used correctly?			3		
Is the number of cases adequate?				2	
Are the experimental methods/ clinical studies adequate?			3		
Is the length appropriate in relation to the content?			3		
Does the reader get new insights from the article?		4			
Please rate the practical significance.			3		
Please rate the accuracy of methods.			3		
Please rate the statistical evaluation and quality control.					1
Please rate the appropriateness of the figures and tables.			3		
Please rate the appropriateness of the references.			3		
Please evaluate the writing style and use of language.		4			
Please judge the overall scientific quality of the manuscript.			3		
Are the methods used worthy of reproduction in greater deal?	No				
Would you be willing to review a revision of this manuscript?	yes				

Comments to author: In this review a novel application of laryngeal adductor reflex (LAR) continuous neuromonitoring (LAR-CIONM) is described and selected applicable references addressed. While very interesting, the review may be improved by more clearly addressing areas of application or specific areas of investigation where LAR-CIONM may provide superior information to established CIONM technologies applied in large series. Furthermore, in some passages efferent and afferent effects of neural compromise appear unclear (p 5; l 47 ff). Comparison of intermittent forms of neuromonitoring with any type of CIONM appears obsolete by the numerous publications referring to its superiority (p7; l45 ff). Precise discrimination of identifying motor and/or sensory decrement of CIONM waves remains to be described (p 9; l 65ff).
Applicability and virtues of LAR-CIONM with regard to thyroid surgery, let alone benefits in comparison to established CIONM technologies are not clearly presented. Thus, either additional data and details may be provided or the conclusions may be adapted..

Reviewer 2: Dionigi, Gianlorenzo

Date received: 13-Aug-2021
Reviewer recommendation: **Return to author for minor modifications**
Reviewer overall scoring: **High**

Assessment Form scores: 5 = High/Yes; 3 = Medium/Adequate; 1 = Low

Is the subject area appropriate for the journal	5				
Does the title clearly reflect the paper's content?	5				
Does the abstract clearly reflect the paper's content	5				
Do the keywords clearly reflect the paper's content?		4			
Does the introduction present the problem clearly?		4			
Are the results/ conclusions justified?		4			
How comprehensive and up-to-date is the subject matter presented?	5				
How adequate is the data presentation?	5				
Are units and terminology used correctly?		4			
Is the number of cases adequate?			3		
Are the experimental methods/ clinical studies adequate?			3		
Is the length appropriate in relation to the content?		4			
Does the reader get new insights from the article?	5				
Please rate the practical significance.		4			
Please rate the accuracy of methods.		4			
Please rate the statistical evaluation and quality control.					1
Please rate the appropriateness of the figures and tables.			3		
Please rate the appropriateness of the references.		4			
Please evaluate the writing style and use of language.		4			
Please judge the overall scientific quality of the manuscript.		4			
Are the methods used worthy of reproduction in greater deal?	Yes				
Would you be willing to review a revision of this manuscript?	Yes				

Comments to author: Thank you for sharing this excellent paper.
It is a privilege and opportunity to review this paper , i.e.: “Continuous intraoperative neuromonitoring of the recurrent laryngeal nerve by eliciting the laryngeal adductor reflex (LAR-CIONM) ".

IN GENERAL

1.This paper is not original as several recent papers in the Literature have measured, reviewed and comment LAR-CIONM methodology, for example:

A: Sinclair CF, Téllez MJ, Ulkatan S. Continuous Laryngeal Adductor Reflex Versus Intermittent Nerve Monitoring in Neck Endocrine Surgery. Laryngoscope. 2021 Jan;131(1):230-236. doi: 10.1002/lary.28710. Epub 2020 May 4. PMID: 32364626.

B: Sinclair CF, Téllez MJ, Sánchez Roldán MA, Ulkatan S. Laryngeal adductor reflex hyperexcitability may predict permanent vocal fold paralysis. Laryngoscope. 2020 Nov;130(11):E625-E627. doi: 10.1002/lary.28477. Epub 2019 Dec 19. PMID: 31854459.

However, LAR-CIONM issue has never been published in Innovative Surgical Sciences Journal to my knowledge.

Furthermore, the topic is important because it is an interesting updated alternative to vagal nerve (VN) stimulation with probe for the C-IONM. Therefore, such publication is relevant in this Journal and could lead to interesting and useful discussions within the scientific community to improve and simplify (!) C-IONM mode of application.

The Authors therefore must be appreciated.

2. The Authors kindly please verify if included text, figures and data sets were previously published. Thank you.

KEYWORDS

“Continuous”, “vocal cord palsy”, “Recurrent laryngeal nerve” terms are missing.

ABSTRACT

The abstract is fine and concise for me.

Line 24: I suggest to modify to insert the explanation for the abbreviation LAR-CIONM. Please modify.

INTRODUCTION

1.Introduction section (first paragraphs) need specific references, thank you.

2.Please notice that there are many abbreviations in the Introduction Section. A summary of abbreviations may be useful for the reader.

3. Line 17, page 4. Please add explanation for CIONM abbreviation. Thank you

4.Personal comment: it might be useful for the Reader to add (A) a schematic figure that represents and explains the mechanism of the LAR. (B) a summary table of all the current methods available today for CIONM and their benefits and limitations. Please notice that there are at least 9 different CIONM modalities reported in the literature till now (Ref: J Invest Surg. 2020. doi: 10.1080/08941939.2020.1734693).

PARAGRAPH “LAR PHYSIOLOGY”

The paragraph is fine with me.

1. Please add a new table to explain R1 and R2 component. I believe this will be useful for the reader.

PARAGRAPH “The evolution and technique of LAR-CIONM”

1.Please add a new table that summarise experimental vs in vivo reports/literature on LAR-CIONM (first Author, year, Journal, n. Patients or NAR). Thank you.

2. Please explain “ETT” and “ISI” abbreviation. Thank you

3. LAR-CIONM is certainly a method that finds application above all when it is difficult to position the CIONM probe on the VN. In this regard, in endoscopic and robotic surgery, the positioning of the CIONM probe on the vagus is difficult. LAR-

CIONM might be a perfect solution. Furthermore, the position of VN is it is extremely varied, sometimes anterior, posterior, between the carotid and jugular (Langenbecks Arch Surg. doi: 10.1007/s00423-010-0693-3. PMID: 20652584).
Add these comments.

FIGURE
Figure 1 seems to appreciate that the EMG tube being used is the commonly used one. Question: Can the technique LAR-CIONM be used on any EMG tube, whether from Inomed, Metrnonic or Langer? Or is it a new dedicated EMG tube? There are compatibility problems?

Figure 2. R1 and R2 (left) need explanation (see above). If possible, please include higher quality figures for the right part of figure 2. Thank you

REFERENCES
References are appropriate, however reference 13 and 14 are the same.

The above concerns should be included in the revised paper.
Thank you for this opportunity to review this paper.
Thank you

Authors' Response to Reviewer Comments

Date received: 24-Feb-2021

Response to reviewer 1

Dear Reviewer 1,

thank you for the time and effort spent on reviewing our manuscript. All of your remarks were considered and included into the revised version. All changes are highlighted in yellow.

Point-by-point revision:

Comments to the Author

The authors address a controversially discussed issue regarding the quality of colorectal cancer in certified multidisciplinary centres.

The approach to compare and discuss single-center data with those from accessible national and international registries is innovative.

The statistical analysis does not seem to be simple. Did the authors have statistical advice?

Statistical advice was given by Prof. Rainer Koch (em.). Medical Faculty Carl Gustav Carus, Technical University Dresden.

How did the authors deal when confidence intervals and standard errors were not available? Please give more details.

Standards errors and confidence intervals were available for the raw SEER-data. The estimation error has been recalculated from the raw material. This enabled us to apply Wald tests allowing an approximate comparison on fixed time points.

For the Munich Cancer Registry data no confidence intervals and standard errors were available, unfortunately even after request no detailed data were provided. Therefore, we were limited to a simple tabular comparison of the publicly accessible relative survival rates by stage. A more detailed information was added to the manuscript's methods (lines 118-120).

It may be discussed whether the results of a single center may represent the general quality of treatment in certified Oncology Centres in Germany. The presented results are excellent but can also just demonstrate the quality of treatment in Hildesheim. It cannot necessarily be related to the certification per se. Ideally, the authors would also provide the corresponding data from Onkozert.

This is a very reasonable comment. We fully agree that it is difficult to prove an effect of certification itself and to distinguish precisely from other interfering factors. We tried to address this problem by following your advice and supplemented the study with OnkoZert data to better classify the observed hospital within other certified centres. We assume that Hildesheim Hospital is a representative certified single centre regarding important certification parameters e.g. the number of primary cases and the complete resection rate. The information is provided in the manuscript's discussion (lines 223 -228).

Please describe the multidisciplinary treatment in Hildesheim more in detail

To the best of our knowledge there is no special treatment or exclusive multidisciplinary approach offered at the hospital compared to other similar institutions. We added a more detailed description of the usual multidisciplinary management in certified cancer centres as required by Onkozert to the manuscript (lines 241-249).

Is there information available for the Munich Cancer Registry regarding data from certified and non-certified centres.

To the best of our knowledge the only publicly available data dealing with this topic is the final evaluation report of the Munich Cancer Registry of 2012 (Schlesinger-Raab A, Schrodi S, Liebetruth E, Meyer R, Schubert-Fritschle G, Engel J. Versorgungsforschung: Kolorektales Karzinom). In this report no significant influence of the centre status on survival could be proven. A further analysis with larger follow-up intervals was suggested.

Please discuss the significant difference in the international comparison of colorectal cancer stage I and II.

We found a significant difference in early stage CRC in favor of the pooled SEER data. As an explanation we think that comorbidities could be a potential confounder as they are not considered in this kind of registry data. It is likely that patients undergoing elective surgery at a certified cancer centre present themselves with more comorbidities compared to smaller non-certified hospitals. Another reason could be a more elaborated level of standardization in the United States. We added this point to the discussion (lines 250-255).

We hope the revision improved the manuscript and that in your opinion it is now appropriate for publication.

Recommendation by the Guest Editor

Revise with minor modifications.

Authors' Response to Reviewer Comments

Date received: 21-Sep-2020

We greatly appreciate the time taken to review this manuscript. Please find detailed responses to each of your queries below.

Response to reviewer 1

Comment 1: In this review a novel application of laryngeal adductor reflecx (LAR) continuous neuromonitoring (LAR-CIONM) is described and selected applicable references addressed. While very interesting, the review may be improved by more clearly addressing areas of application or specific areas of investigation where LAR-CIONM may provide superior information to established CIONM technologies applied in large series.

Response 1:

We thank you for your kind review of this manuscript.

The LAR is becoming a important method for reducing laryngeal complications and enhancing safety during resection of complex posterior fossa and brainstem lesions, vagus nerve schwannomas, and thyroid and parathyroid masses.

In neck endocrine surgeries, the LAR-CIONM is used to monitor the RLN, which is the main nerve at risk of injury; therefore, its utility is comparable to other existing methodologies with regards to outcome measures. The advantage of LAR-CIONM over CMAP-CIONM using a vagal electrode is the avoidance of dissection of the vagus nerve for electrode placement – LAR-CIONM is solely reliant on placement of a monitored EMG endotracheal tube. We have expanded the discussion of these points in the paper in the neck endocrine surgery section.

For other surgeries where the vagus nerve is at risk of injury, LAR-CIONM is a major breakthrough to assess the integrity of both sensory and motor functions of laryngeal and vagus nerves, and intrinsic brainstem pathways and nuclei. For the first time, sensory vagal fibers in the IBSLN can be monitored continuously during relevant surgeries, thereby providing a methodology that can prevent laryngopharyngeal sensory impairments, the clinical results of which can be devastating for patients as they relate to swallowing and airway protective functions. We have attempted to highlight this in the relevant sections on LAR-CIONM.

The LAR allows nuances in laryngeal behavior to be uncovered. For instance, normal postoperative vocal fold motility while the patient says "eee" under laryngoscope examination does not exclude laryngeal hypoesthesia and therefore does not rule out a higher risk of aspiration. Another nuance is that provides evidence that assessment of voice and speech using normal vocal fold mobility as a marker for normal function is insufficient for entirely assessing patient's outcome, because automatic functions such as swallowing, coughing, and aspiration have been demonstrated to occur through separate pathways than voluntary functions such as speech. We have added a paragraph about this in the neck endocrine section of the paper.

The LAR is stable and bilateral under total intravenous anesthesia. However, under inhalational halogenated anesthetic agents, the LAR weakness. This concept has important implications for managing general anesthesia and preventing anesthesia-related aspiration events. Preservation of the LAR could be desirable for patients at high risk of aspiration, such as following emergency intubation where preoperative fasting was impossible. Conversely, patients at high risk of laryngospasm where elimination of the LAR is desirable would benefit from administering inhalational agents immediately before extubation with or without topical anesthesia applied directly to supraglottic mucosa. We have added a new section (Anesthetic implications) to the paper, just prior to the conclusion.

Comment 2: Furthermore, in some passages efferent and afferent effects of neural compromise appear unclear (p 5; l 47 ff).

Response 2:

Monitoring with the LAR help surgeons discern sensory injuries from motor injuries and predict laryngeal function outcomes. Monitoring the sensory component of the vagus nerve with the LAR is useful not only when dealing with the peripheral nerves, but also in posterior fossa and brainstem surgeries as it can detect laryngeal mucosa hypoesthesia and anticipating possible swallowing dysfunction.

In neck endocrine surgeries, only motor fibers of the vagus nerve via the RLN are at risk of injury. However, if there is a

tumor involving the vagus nerve (proximal to the exit of the superior laryngeal nerve), or in the posterior fossa, both sensory and motor fibers of the affected vagus nerve are at risk of injury. As depicted in the new figure (Figure 2), by selecting the side of stimulation and recording of the endotracheal tube electrodes for eliciting the LAR, this technique differentiates sensory from motor involvement. This new figure is referenced in the text and a new discussion of how to distinguish efferent from afferent fibers added to the paper under the heading "Choice of side for stimulation versus recording during LAR-CIONM".

Electrical stimulation of the laryngeal mucosa on the side contralateral to the tumor carries sensory information to the brainstem via the left internal superior laryngeal nerve (iSLN), and activation of motor neurons located on both nucleus ambiguus will follow. By recording the contraction of vocal muscles on the right side (ipsilateral to the tumor), the LAR provides data on the motor integrity of the vagus nerve at risk, so the LAR is called motor-LAR. Panel B: Stimulation of the laryngeal mucosa ipsilateral to the tumor provides sensory information of the vagus nerve at risk; by the iSLN on the right side, activation of motor neurons located on both nucleus ambiguus will follow. The LAR recorded on the contralateral vocal fold muscle is then called sensory-LAR.

Comment 3: Precise discrimination of identifying motor and/or sensory decrement of CIONM waves remains to be described

Response 3: Stimulating the laryngeal mucosa contralateral to the tumor and recording the LAR ipsilateral to the tumor provides data on the motor integrity of the vagus nerve at risk, so the LAR is called motor-LAR. Stimulating the laryngeal mucosa ipsilateral to the tumor provides sensory information of the vagus nerve at risk. This evoked LAR is called sensory-LAR and is recorded on the contralateral vocal fold muscle (Figure xx). Both sensory and motor divisions of the LAR are assessed alternatively during surgery. If LAR amplitude decreases ≥ 50% from baseline the surgeon is warned. A permanent loss of the sensory-LAR or motor-LAR predicates for laryngeal hypoesthesia or vocal fold paralysis, respectively. Discriminating between sensory and motor vagal nerve fibers using LAR-CIONM and warning criteria have been described, although a large scale trial is needed to confirm these pilot study findings (see text and references). This has been added to the text.

Comment 4: Applicability and virtues of LAR-CIONM with regard to thyroid surgery, let alone benefits in comparison to established CIONM technologies are not clearly presented. Thus, either additional data and details may be provided or the conclusions may be adapted. We have added paragraphs to the neck endocrine section detailing advantages as discussed in prior responses.

Response to reviewer 2

Comment 1: 1.This paper is not original as several recent papers in the Literature have measured, reviewed and comment LAR-CIONM methodology, for example:

A: Sinclair CF, Téllez MJ, Ulkatan S. Continuous Laryngeal Adductor Reflex Versus Intermittent Nerve Monitoring in Neck Endocrine Surgery. Laryngoscope. 2021 Jan;131(1):230-236. doi: 10.1002/lary.28710. Epub 2020 May 4. PMID: 32364626.

B: Sinclair CF, Téllez MJ, Sánchez Roldán MA, Ulkatan S. Laryngeal adductor reflex hyperexcitability may predict permanent vocal fold paralysis. Laryngoscope. 2020 Nov;130(11):E625-E627. doi: 10.1002/lary.28477. Epub 2019 Dec 19. PMID: 31854459.

However, LAR-CIONM issue has never been published in Innovative Surgical Sciences Journal to my knowledge.

Furthermore, the topic is important because it is an interesting updated alternative to vagal nerve (VN) stimulation with probe for the C-IONM. Therefore, such publication is relevant in this Journal and could lead to interesting and useful discussions within the scientific community to improve and simplify (!) C-IONM mode of application.

The Authors therefore must be appreciated.

Response 1:

Thank you very much for your appreciation and insights. This was an invited paper designed to review LAR-CIONM utility. We appreciate you taking the time to read this review.

Comment 2: 2.The Authors kindly please verify if included text, figures and data sets were previously published. Thank you.

Figures 1 and 3 are original. The newly added Figure 2 is reproduced from the book: Neurophysiology in Neurosurgery (2nd Edition, 2020) A Modern Approach. Elsevier Inc. Editors: Vedran Deletis,Jay Shils, Francesco Sala and Kathleen Seidel. ISBN 978-0-12-815000-9 with permission. This is a review paper and thus data sets reported have been previously published and are referenced in the text.

Comment 3: KEYWORDS

“Continuous”, “vocal cord palsy”, “Recurrent laryngeal nerve” terms are missing.

Response 3: Thank you for your comment. These keywords have been added.

Comment 4: ABSTRACT

The abstract is fine and concise for me.

Line 24: I suggest to modify to insert the explanation for the abbreviation LAR-CIONM. Please modify

Response 4:

Thank you for pointing out this lack of clarity. The abbreviation has been explained in the manuscript as follows:

This methodology involves continuous intraoperative neuromonitoring of the laryngeal adductor reflex and is thus termed 'LAR-CIONM'

Comment 5: INTRODUCTION 1.Introduction section (first paragraphs) need specific references, thank you.

Response 5: Thank you for highlighting this issue. Specific references have been added in the INTRODUCTION section.

Comment 6: INTRODUCTION 2.Please notice that there are many abbreviations in the Introduction Section. A summary of abbreviations may be useful for the reader.

Response 6:

Thank you. The following list of abbreviations has been added in the manuscript before INTRODUCTION section.

LAR: laryngeal adductor reflex; IONM intraoperative neuromonitoring; CIONM: continuous intraoperative neuromonitoring;

LAR-CIONM: continuous intraoperative neuromonitoring of the laryngeal adductor reflex; iSLN: internal branch of the

superior laryngeal nerve; RLN: recurrent laryngeal nerve; EMG: electromyographic; ms: milliseconds; CMAP: compound

muscle action potential; R1: short-latency component of the laryngeal adductor reflex; R2 long-latency component of the laryngeal adductor reflex; TIVA: total intravenous anesthesia; ETT; endotracheal tube.

Comment 7: INTRODUCTION 3. Line 17, page 4. Please add explanation for CIONM abbreviation. Thank you

Response 7: Thank you for your comment. The sentence has been rephrased as follows:

‘These investigations have enabled the LAR to be used as a continuous intraoperative neuromonitoring technique (CIONM) for the RLN and vagus nerves, thus this methodology is termed 'LAR-CIONM'

Comment 8: 4.Personal comment: it might be useful for the Reader to add (A) a schematic figure that represents and explains the mechanism of the LAR. (B) a summary table of all the current methods available today for CIONM and their benefits and limitations. Please notice that there are at least 9 different CIONM modalities reported in the literature till now (Ref: J Invest Surg. 2020. doi: 10.1080/08941939.2020.1734693).

Response 8:

A schematic figure that represents and explains the mechanism of the LAR from the book:

Neurophysiology in Neurosurgery (2nd Edition, 2020) A Modern Approach. Elsevier Inc. Editors: Vedran Deletis,Jay Shils, Francesco Sala and Kathleen Seidel. ISBN 978-0-12-815000-9 has been added to the figures (Figure 2). A table has also been added (Table 1).

Author	Targete d structur e	CIONM methods	Invasive	Motor monitoring	Sensory monitoring	Brainstem (medulla) monitoring	Setting (number of nerves)
Lamade W, 1997	RLN	Trans-tracheal by endotracheal tube	No	Yes	No	No	Experimental
Smith DB, 1989	RLN	Double-ballooned endotracheal tube and pressure transducer system	No	Yes	No	No	Experimental
Lamade W, 2007<br>Ulmer C, 2008<br>Schneider R, 2009	VN	Vagal nerve cuff electrode	Yes	Yes	No	No	Experimental<br>In-vivo (n=19)<br>In-vivo (n=78)
Chiang FY, 2015<br>Zhang D, 2019	RLN	Stimulating dissecting instruments	No	Yes	No	No	In-vivo (n=168)<br>Experimental
Sinclair CF, 2020<br>Tellez MJ, 2020	VN and Medulla	Endotracheal tube electrodes	No	Yes	Yes	Yes	In-vivo (n=205)<br>In-vivo (n=53)
Liu XL, 2016	RLN	hand probe continuous stimulation	No	Yes	No	No	In-vivo(n=120)
Wu CW, 2013	VN	hand probe continuous stimulation on the carotid sheath	No	Yes	No	No	In-vivo (n=376)
Zhang D, 2018	VN	Percutaneous stimulation	Yes	Yes	No	No	In-vivo (n=277)
Suh I, 2016	VN	Transcutaneous stimulation	Yes	Yes	No	No	In-vivo(n=4)

Add references

Lamade W,Meyding-Lamade U, Hund E, Senninger N, Herfarth C.[Transtracheal monitoring of the recurrent laryngeal nerve.Prototype of a new tube]. Chirurg. 1997;68(2):193–195. FebGerman

Smith DB, Woody EA, Richardson M, Olsen HL, 3rd, Blakeslee DB. A device for the intraoperative identification of the recurrent laryngeal nerve in piglets. Otolaryngol Head Neck Surg. 1989;100(2):137–145

Lamade W, Ulmer C, Seimer A, et al. Anew system for continuous recurrent laryngeal nerve monitoring. Minim Invasive Ther Allied Technol. 2007;16(3):149–154.

Ulmer C, Koch KP, Seimer A, et al. Real-time monitoring of the recurrent laryngeal nerve: an observational clinical trial. Surgery. 2008;143(3):359–365. doi:10.1016/j.surg.2007.10.007.

Schneider R, Przybyl J, Hermann M, Hauss J, Jonas S, Leinung S. A newanchorelectrode design for continuous neuromonitoring of the recurrent laryngeal nerve byvagal nerve stimulations. Langenbecks Arch Surg. 2009;394(5):903–910. doi:10.1007/s00423-009-0503-y.

Chiang FY, Lu IC, Chang PY, et al. Stimulating dissecting instruments during neuromonitoring ofRLN in thyroid surgery. Laryngoscope. 2015;125(12):2832–2837. doi:10.1002/lary.25251.

Zhang D, Li S, Dionigi G, et al. Stimulating and dissecting instrument for transoral endoscopic thyroidectomy: proof of concept investigation. SurgEndosc. 2019;34:996–1005. doi:10.1007/s00464-019-06936-2.

Liu XL, Wu CW, Zhao YS, et al. Exclusive real-time monitoring during recurrent laryngeal nerve dissection in conventional monitored thyroidectomy. Kaohsiung J Med Sci. 2016;32(3):135–141. doi:10.1016/j.kjms.2016.02.004.

Wu CW, Dionigi G, Chen HC, et al. Vagal nerve stimulation without dissecting the carotid sheath during intraoperative neuromonitoring of the recurrent laryngeal nerve in thyroid surgery. Head Neck. 2013;35(10):1443–1447. doi:10.1002/hed.23154.

Zhang D, Fu Q, Dionigi G, et al. Intraoperative neural monitoring in endoscopic thyroidectomy via bilateral areola approach. Surg Laparosc Endosc Percutan Tech. 2018;28(5):303–308. doi:10.1097/SLE.0000000000000542.

Suh I, Yingling C, Randolph GW, Duh QY. A novel method of neuromonitoring inthyroidectomy and parathyroidectomy using transcutaneous intraoperative vagal stimulation. JAMA Surg. 2016;151(3):290–292. doi:10.1001/jamasurg.2015.3249

Comment 9: PARAGRAPH “LAR PHYSIOLOGY”

The paragraph is fine with me.

1. Please add a new table to explain R1 and R2 component. I believe this will be useful for the reader.

Answer to Comment 9

?????

Comment 10: PARAGRAPH “The evolution and technique of LAR-CIONM”

1.Please add a new table that summarise experimental vs in vivo reports/literature on LAR-CIONM (first Author, year, Journal, n. Patients or NAR). Thank you.

Answer to Comment 10

Catherine, use same table as comment 8

Comment 11: 2. Please explain “ETT” and “ISI” abbreviation. Thank you

Answer to Comment 11

Thank you for highlighting this issue. ETT abbreviation has been explained in the text and added in the abbreviation list. The ISI abbreviation has been explained and the abbreviation eliminated.

Comment 12: 3. LAR-CIONM is certainly a method that finds application above all when it is difficult to position the CIONM probe on the VN. In this regard, in endoscopic and robotic surgery, the positioning of the CIONM probe on the vagus is difficult. LAR-CIONM might be a perfect solution. Furthermore, the position of VN is it is extremely varied, sometimes anterior, posterior, between the carotid and jugular (Langenbecks Arch Surg. doi: 10.1007/s00423-010-0693-3. PMID: 20652584). Add these comments.

Answer to Comment 12

Thank you for your suggestion. The paragraph has been added in the manuscript.

Comment 13: FIGURE

Figure 1 seems to appreciate that the EMG tube being used is the commonly used one. Question: Can the technique LAR-CIONM be used on any EMG tube, whether from Inomed, Metrnonic or Langer? Or is it a new dedicated EMG tube? There are compatibility problems?

Answer to Comment 13

Thank you for this interesting question.

Structural elements differ between the commercially available endotracheal tubes resulting in unique advantages and disadvantages for LAR stimulation and recording. We have tried different available tubes, all of them are capable of eliciting the LAR, however none of them is optimal.

To date we have found a significantly higher LAR amplitude using the NIM-EMG-ETT compared to TriVantage tube, for the Medtronic group. For the INOMED endotracheal tube, a customized connector provided by the manufacturer was used to connect the eight contact electrodes separately. We do not have experience with the Langer electrode.

One vital key to obtain an adequate LAR rests on choosing the correct size of the tube according to the patient's laryngeal anatomy.

Comment 14: Figure 2. R1 and R2 (left) need explanation (see above). If possible, please include higher quality figures for the right part of figure 2. Thank you

Answer to Comment 14

????

Comment 15: REFERENCES

References are appropriate, however reference 13 and 14 are the same.

Answer to Comment 15

Comments by the Editor-in-Chief to Revised Submission

All reviewer comments were addressed adequately and the manuscript should be published in its present stage.
